# Laser and radiofrequency for treating genitourinary syndrome of menopause in breast cancer survivors: A systematic review of randomized controlled trial

**DOI:** 10.1002/ijgo.70665

**Published:** 2025-11-15

**Authors:** Nicoli Serquiz, Ayane Cristine Alves Sarmento, Antonio Carlos Queiroz de Aquino, Natalie Rios Almeida, Maria Luísa Nobre, Juliana Dantas de Araújo Santos Camargo, Kleyton Santos de Medeiros, Ana Katherine Gonçalves

**Affiliations:** ^1^ Postgraduate Program Student in Health Science Federal University of Rio Grande Do Norte (UFRN) Natal Brazil; ^2^ Department of Obstetrics and Gynecology Federal University of Rio Grande Do Norte (UFRN) Natal RN Brazil; ^3^ Department of Clinical Analysis and Toxicology Federal University of Rio Grande Do Norte (UFRN) Natal RN Brazil; ^4^ Institute of Education, Research and Innovation League against Cancer Natal RN Brazil; ^5^ Faculty of Health Sciences of Trairí (FACISA) Federal University of Rio Grande Do Norte (UFRN) Santa Cruz RN Brazil; ^6^ Postgraduate Program in Obstetrics and Gynecology State University of Campinas (UNICAMP) Campinas Brazil; ^7^ Department of Surgery Federal University of Rio Grande Do Norte (UFRN) Natal RN Brazil

**Keywords:** atrophy, breast neoplasms, cancer survivors, genitourinary syndrome of menopause (GSM), laser therapy, radiofrequency therapy

## Abstract

**Background:**

Breast cancer survivors (BCS) often experience more severe symptoms of genitourinary syndrome of menopause (GSM). As estrogen‐based hormonal therapy is generally avoided in BCS, physical energy methods may offer promising non‐hormonal alternatives. However, the efficacy and safety of these treatments remain controversial.

**Objectives:**

To evaluate the efficacy and safety of physical energy methods (laser and radiofrequency) for treating GSM in BCS.

**Search Strategy:**

The databases PubMed/MEDLINE, Embase, Scopus, Web of Science, Cochrane Library, SciELO, LILACS, and Clinical Trial databases were searched from their inception to July 2024, with no language restrictions.

**Selection Criteria:**

We included randomized controlled trials (RCTs) that assessed the efficacy and safety of any physical energy methods for treating GSM in BCS.

**Data Collection and Analysis:**

Two authors independently selected studies based on titles, abstracts, and full texts to meet the inclusion criteria. Due to heterogeneity in methodologies and outcomes, a meta‐analysis was not possible, so a narrative synthesis was conducted. Data were extracted, and the risk of bias was assessed using the Cochrane risk‐of‐bias tool (RoB 2). The Grading of Recommendations, Assessment, Development, and Evaluation (GRADE) approach was used to assess the strength of the evidence.

**Main Results:**

Three RCTs involving 185 participants, and three different physical methods (microablative fractional CO_2_ laser, erbium photothermal yttrium‐aluminum‐garnet laser, and radiofrequency) met the inclusion criteria for this systematic review. Laser and radiofrequency treatments improved GSM symptoms in BCS, with improvements in dyspareunia, the Vaginal Health Index, and quality of life in the short term, with minimal adverse events. Overall, only one study had a low risk of reporting bias, whereas two studies raised concerns due to critical weaknesses. Confidence in the evidence is low and critical across all studies.

**Conclusions:**

Physical energy methods show short‐term safety in treating GSM in BCS. However, limited blinded clinical trials result in uncertain efficacy.

## 1 INTRODUCTION

Over the past few decades, women have been surviving longer after a cancer diagnosis, making it increasingly important to address their health needs. Over 70% of breast cancer survivors (BCS) are expected to experience symptoms of genitourinary syndrome of menopause (GSM), a common and yet underdiagnosed condition in this population.^1^, ^2^

Vulvovaginal atrophy is a key component of GSM, with common symptoms including genital dryness, burning, dyspareunia, irritation, sexual dysfunction, and urinary issues such as urgency, dysuria, and recurrent urinary tract infections. These symptoms often worsen in BCS compared with postmenopausal women. Meriggiola et al.^3^ observed that women with breast cancer more frequently presented severe vulvovaginal atrophy (41.2% versus 25.4%) and greater symptom burden, particularly vulvar dryness, pain, and sexual distress with a stronger impact on emotional well‐being. Cancer therapies such as chemotherapy, tamoxifen, and aromatase inhibitors exacerbate a hypoestrogenic state, reducing circulating estrogen levels in genital tissues, which can negatively impact sexual function and significantly affect quality of life (QoL).^4^, ^5^

Given the controversy surrounding estrogen‐based treatments, non‐hormonal therapies like lubricants and vaginal moisturizers are typically the first‐line choice.^6^ However, these options offer only mild efficacy, usually providing short‐term relief without addressing urogenital aging.^7^, ^8^ Currently, there is no consensus on how to treat moderate to severe GSM in BCS.^9^, ^10^

Intravaginal physical energy methods have emerged as a non‐pharmacologic therapeutic option for managing GSM in recent years. Techniques such as microablative fractional CO_2_ laser, erbium photothermal yttrium‐aluminum‐garnet (Er:YAG) laser, and radiofrequency represent a novel approach within energy‐based therapies. Meta‐analyses and recent reviews have shown promising results in improving sexual health, alleviating GSM symptoms, and restoring vaginal health in postmenopausal women.^11^, ^12^, ^13^

A previous meta‐analysis of non‐randomized studies suggested that vaginal laser therapy was effective, showing improvements in the Vaginal Health Index (VHI), Visual Analogue Scale (VAS) scores for dyspareunia and vaginal dryness, and sexual function.^14^ However, the evidence for the true effectiveness of these therapies remains limited, as additional systematic reviews have reached similar conclusions, relying mainly on observational studies and short follow‐up periods, and have provided low‐certainty evidence.^15^, ^16^ Therefore, a systematic review of randomized controlled trials (RCTs) offering the highest level of evidence is needed. This systematic review of RCTs aimed to evaluate the efficacy and safety of all currently available physical energy therapies for relieving the signs and symptoms of GSM in BCS.

## 2 MATERIALS AND METHODS

This systematic review was conducted following the Cochrane Handbook for Systematic Reviews of Interventions^17^ and reported following the Preferred Reporting Items for Systematic Reviews and Meta‐Analyses (PRISMA) statement.^18^ The review was prospectively registered in the International Prospective Registry of Systematic Reviews (PROSPERO) database (CRD42023387680) and the protocol was previously published.^19^

### 2.1 Information sources

A review was conducted to answer the question: “Are intravaginal physical energies effective and safe for treating GSM in BCS?” Using the PICOS framework: Population/Participants: BCS with GSM; Intervention: intravaginal physical energy (laser or radiofrequency); Comparison: no treatment, placebo, sham, or other treatments; Outcome: improvements in vaginal atrophy symptoms (dryness, dyspareunia, itching, burning), sexual dysfunction, dysuria, urinary symptoms (incontinence, urgency, and frequency), vaginal health, and adverse events; and Study design: randomized clinical trials.

### 2.2 Search strategy

A comprehensive search of bibliographic databases and gray literature (Google Scholar) was conducted in July 2024 under the guidance of an experienced librarian (DMSS—UFRN, Natal, Brazil), following systematic review and meta‐analysis guidelines (Appendix S1). The databases searched included PubMed/MEDLINE, Embase, Scopus, Web of Science, Cochrane Central Register of Controlled Trials, Scientific Electronic Library Online (Scielo), Latin America and the Caribbean Health Sciences Literature (LILACS), and clinical trial databases (www.trialscentral.org, www.controlled‐trials.com, and ClinicalTrials.gov). No language or date restrictions were imposed. A combination of Medical Subject Heading (MeSH) terms and keywords was used in the searches. The complete search strategy for the PubMed database is provided in Table 1.

**TABLE 1:** PubMed search strategy.

Number	Search item
1	Breast neoplasm
2	Breast cancer
3	Female cancer
4	Cancer survivors
5	Menopause
6	Postmenopause
7	OR/1–6
8	Laser therapy
9	Lasers
10	Erbium YAG laser
11	Lasers, CO_2_
12	Laser, Carbon dioxide
13	Radiofrequency therapy
14	Radiofrequency therapies
15	Therapy, radiofrequency
16	Radio‐frequency therapy
17	Energy‐based devices
18	OR/8–17
19	Therapeutics
20	Therapeutic
21	Therapy
22	Therapies
23	Treatment
24	Treatments
25	OR/19–24
26	Genitourinary syndrome of menopause
27	Atrophic vaginitis
28	Atrophy
29	Dyspareunia
30	Sexual health
31	Sexual dysfunctions, physiologic
32	Dysuria
33	Quality of life
34	OR/26–33
34	7 AND 18 AND 25 AND 34

### 2.3 Eligibility criteria

As inclusion criteria, RCTs evaluating the efficacy and safety of any intravaginal physical energy methods (laser and radiofrequency) for treating GSM in non‐metastatic BCS who had completed primary cancer treatment, regardless of age, and who were still sexually active or willing to resume sexual activity with one or more GSM symptoms were included. Studies were excluded if they involved women with recurrent or metastatic cancer, previous reconstructive pelvic surgery involving a mesh for prolapse, or active genital infections, or if they did not assess the relevant outcomes. Observational studies, case reports, review articles, and case series were also excluded.

### 2.4 Study selection

Following standardized database searches, the retrieved literature was imported into Rayyan software (Mourad Ouzzani, University of Oxford, UK), and duplicates were removed. Two authors (ACAS and NS) independently selected studies based on titles and abstracts, followed by full‐text reviews. Any disagreements were resolved by a third author (AKG).

### 2.5 Data extraction and management

The authors developed and tested a standardized data extraction form. Three authors (ACAS, NS, and KSM) independently extracted the following data: author, publication year, search date, country, study design, mean age, sample size, follow‐up duration, type of intravaginal physical energy used, therapeutic protocol, hormonal adjuvant therapy, and outcomes. In cases of missing data, the corresponding authors were contacted via email. Any disagreements were resolved through consensus discussions among the authors, with a fourth author (AKG) reviewing the final decisions.

### 2.6 Outcome measures

The primary outcome was improvement of GSM symptoms, including: vaginal atrophy (dryness, dyspareunia, itching, and burning), which was assessed using the VAS^20^; sexual symptoms assessed by the Female Sexual Function Index (FSFI)^21^; and vaginal health, which was evaluated using the VHI, including epithelial integrity, vaginal elasticity, moisture, fluid volume, and vaginal pH.^22^

Secondary outcomes included the following: frequency, urgency, nocturia, and urinary incontinence; Vaginal Maturation Index (VMI), including epithelial maturation and maturation value; QoL; and adverse events.

### 2.7 Assessment of risk of bias

The risk of bias in the included studies was independently assessed by two authors (NS and ACAS) using the Cochrane risk‐of‐bias tool (RoB 2).^23^ The RoB 2 tool evaluates bias across five domains: randomization process, deviations from intended interventions, missing outcome data, outcome measurement, and selection of reported results. Each domain includes signaling questions that guide judgments of “low risk of bias”, “some concerns”, or “high risk of bias”.^17^, ^23^

### 2.8 Data synthesis

Due to the high heterogeneity among the studies, on outcomes, control groups, and interventions, a meta‐analysis could not be performed. Instead, we provide a narrative description of the characteristics and findings of the included studies.

### 2.9 Certainty assessment

The certainty of evidence was graded using the Grading of Recommendations, Assessment, Development, and Evaluation (GRADE) approach. The assessment summary incorporates broader measures to ensure judgments on the risk of bias, consistency, objectivity, and accuracy. The quality of evidence was evaluated based on factors such as risk of bias, indirectness, inconsistency, imprecision, and publication bias.^24^

## 3 RESULTS

### 3.1 Study selection

**FIGURE 1:**
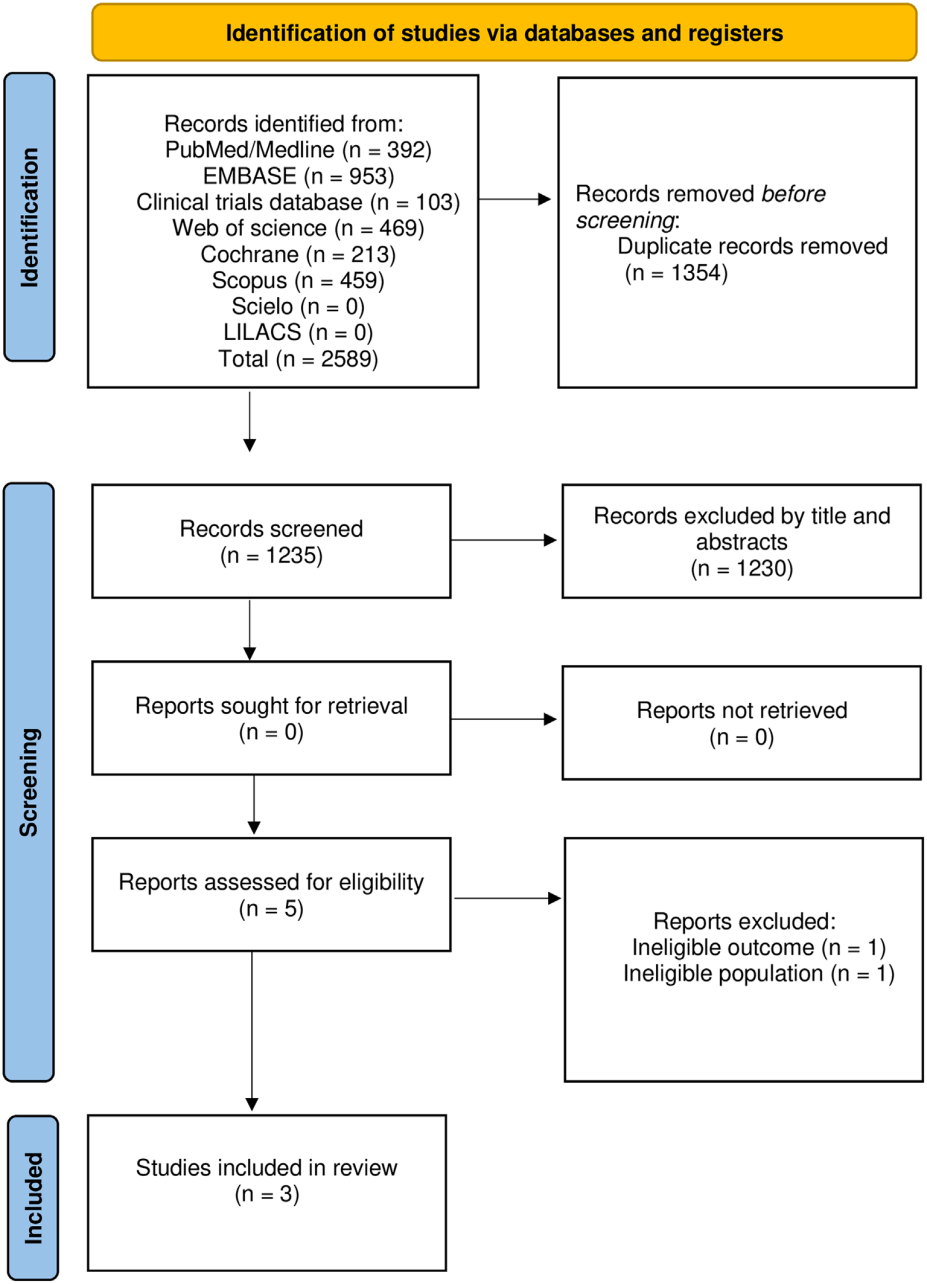
Prisma flow chart overview of study.

A total of 2588 articles were retrieved from the databases. After removing duplicates, 1235 records were screened based on their titles and abstracts, resulting in five articles selected for full‐text analysis. Ultimately, three RCTs met the eligibility criteria and were included in the final review. The details of the selection process are illustrated in Figure 1.

During the selection phase, seven trials were identified as ongoing. The full list of these trials is provided in Table S1.

### 3.2 Study characteristics

A total of 185 patients were included in this review, with a mean age of 51–56.6 years. The three RCTs were published in 2023 and were conducted in Austria,^25^ Brazil,^26^ and Spain.^27^ Most of the participants were receiving adjuvant hormone therapy—174 (94%), with aromatase inhibitors (77%) or tamoxifen (17%). Among the three included RCTs, Fernandes et al.^26^ examined both CO_2_ laser and radiofrequency, whereas Mension et al.^27^ investigated only CO_2_ laser, and Gold et al.^25^ evaluated the Er:YAG laser.

The interventional compared groups were a sham laser,^27^ promestriene,^26^ and hormone‐free vaginal suppository.^25^ The general characteristics of the included studies are presented in Table 2.

**TABLE 2:** Characteristics of the randomized controlled trials included.

Author (year) Country	Study design	Intervention type	Control	Therapeutic protocol	Sample size	Age, years		Follow up, months	Hormonal BC therapy	Outcome measures
						Interven tion	Control			
Mension et al. (2023)^27^ Spain	RCT, double‐blind	CO_2_L	Sham laser	First‐line therapy (moisturizer + vaginal vibrator), five laser therapies monthly	72	51.3^a^	53.7	0–6	AI = 72	**Primary:** FSFI **Secondary:** VAS; Body image; Quality of life; VHI; Vaginal pH, VMI; Vaginal epithelium thickness; Vaginal epithelium elasticity/Adverse effects and tolerance
Fernandes et al. (2023)^26^ Brazil	RCT, not blinded	CO^2^L RF	Promestriene	Three physical therapies monthly (CO_2_L and RF) Promestriene: 10 mg daily for 21 days, followed 2 non‐consecutive days per week for 4 months	70	52.43 (CO_2_L)^a^ 51 (RF)^a^	56.65	0–4	TAM = 25 AI = 45	**Primary:** VAS **Secondary:** Epithelium: thickness, hyperkeratosis, dermal papillae and Stroma: number of vessels
Gold et al. (2023)^25^ Austria	RCT, single‐blind	Er: YAG	Suppository hormone‐free	Two laser therapies monthly; suppository vaginally daily for 10 days, and then every third day	43	54^b^	56	0–3	AI = 25 TAM = 7 None = 10	**Primary:** VHI **Secondary:** Subjective urogenital atrophy and degree of discomfort or pain; PGI‐I and PGI‐S; Quality of life; Sexual health problems; Pelvic floor symptoms and Treatment satisfaction

Abbreviations: AI, aromatase ihnibitor; CO_2_L, thermoablative fractional CO_2_ laser; FSFI, Female Sexual Function Index; PGI‐I, Patient Global Impression of Improvement; PGI‐S, Patient Global Impression of Severity; RCT, randomized clinical trial; RF, radiofrequency; TAM, tamoxifen; VAS, Visual Analog Scale; VHI, Vaginal Health Index; VMI, Vaginal Maturation Index; YAG laser, YAG, Yttrium‐aluminum‐garnet laser.

^a^ Mean.

^b^ Median.

### 3.3 Risk of bias of included studies

**FIGURE 2:**
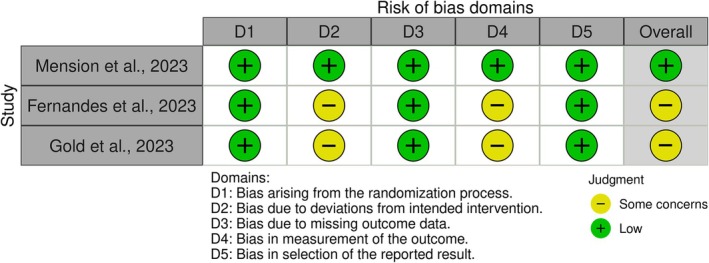
Risk of bias of included clinical trials.

Overall, one study had a low risk of bias,^27^ and two had some concerns,^25^, ^26^ because of a lack of clarity about the process of randomization and blinding (Figure 2).

### 3.4 Synthesis of results

All three studies reported improvements in dyspareunia with physical energy methods. Dyspareunia was consistently measured using a numerical scale ranging from 0 to 10. Mension et al.^27^ observed a reduction in dyspareunia severity from a mean ± standard deviation (SD) score of 7.3 ± 2.4 to 3.0 ± 2.8. Fernandes et al.^26^ found improvements with laser treatment, with scores changing from a mean ± SD of 7.27 ± 3.65 to 2.71 ± 3.4, and with radiofrequency treatment, scores changed from 7.7 ± 3.78 to 4.07 ± 4.08. Gold et al.^25^ reported an improvement in dyspareunia and vaginal dryness, with scores of dyspareunia decreasing from a median (interquartile range [IQR]) of 9 (IQR 8–10) to 7 (IQR 1–9) (*P* = 0.002) and for vaginal dryness a mean of 7 (IQR 3–8) to 3 (IQR 0–7) (*P <* 0.001) in the Er:YAG Laser group (LG). However, no significant differences in mean outcomes were observed when comparing the groups.

With respect to vaginal health, as measured by the VHI, two studies demonstrated significant improvements following treatment. Mension et al.^27^ found that VHI scores increased significantly in both the CO_2_ laser and sham groups, with no statistically significant difference between them (*P* = 0.17). Likewise, Gold et al.^25^ observed significant improvements in both the Er:YAG laser and hyaluronic acid groups (*P* = 0.001), although no significant difference was detected between treatments (*P* = 0.232).

In relation to atrophy, two studies evaluated histologic parameters through vulvovaginal biopsy. Fernandes et al.^26^ found an increase in epithelial thickness in both energy‐based treatment groups (CO_2_ laser: mean ± SD, 2.36 ± 0.87 μm to 2.68 ± 0.94 μm; radiofrequency: 2.52 ± 0.80 μm to 2.75 ± 1.25 μm) compared with the control group; however, the difference was not statistically significant (*P* = 0.73), which may be attributed to the low proportion of patients presenting histologic atrophy (5.71%). Likewise, Mension et al.^27^ reported an increase in vaginal epithelial thickness after laser treatment (0.089 ± 0.062 mm to 0.110 ± 0.049 mm), with no significant between‐group difference in mean change (−0.019 ± 0.018; *P* = 0.30).

Only one study evaluated sexual function, using both the FSFI and VMI indices. Mension et al.^27^ observed an increase in FSFI scores in the laser group (mean ± SD: 14.8 ± 8.8 to 20.0 ± 9.5), along with a corresponding improvement in the VMI (7.9% ± 17.6% to 18.1% ± 19.2%). Nevertheless, the between‐group differences in mean change were not statistically significant (*P* = 0.15).^27^

Gold et al.^25^ was the only study to report on urinary incontinence symptoms, with no treatment arm demonstrating complete resolution of these symptoms.

Among the included trials, two provided data on QoL. Only Gold et al.^25^ reported improvements in QoL domains based on the European Organization for Research and Treatment of Cancer quality of life questionnaire (EORTC QLQ‐BR45) questionnaire. When comparing the groups, no significant differences were observed in all QoL domains. Mension et al.^27^ evaluated quality of life by the Short Form 12, with no differences in improvements (−0.3 ± 3.6 versus −0.7 ± 3.2; *P* = 0.39).

Fernandes et al.^26^ reported that the most common adverse effect was pain during CO_2_ laser or radiofrequency application, with no clinical or histology melanoses or any form of tissue damage. Mension et al.^27^ related complications in 13 patients (37%) in the laser group and 10 patients (27%) in the sham group, with no differences in severity or incidence between groups. No adverse effects were reported by Gold et al.^25^ No severe adverse events were reported in any of the RCTs, and none of the participants discontinued the treatment.^25^, ^26^, ^27^

The certainty of evidence for each outcome assessed in the RCTs was classified according to GRADE guidelines, as detailed in Table 3. Most GRADE ratings for dyspareunia, vaginal health, and QoL were low or very low.

**TABLE 3:** GRADE certainty of evidence.

Certainty assessment							Impact	Certainty	Importance
No. of studies	Study design	Risk of bias	Inconsistency	Indirectness	Imprecision	Other considerations			
Dyspareunia (follow up: mean 6 months; assessed with: VAS/NRS)									
3	Randomized trials	Serious^a^	Very serious^b^	Not serious^c^	Not serious	None	Physical energy (Laser and radiofrequency) improved in symptom severity; however, no differences in the mean improvement between control groups	⨁◯◯◯ Very low	Crítical
Vaginal health (follow up: mean 6 months; assessed with: VHI)									
2	Randomized trials	Serious^a^	Serious^b^	Not serious	Not serious	None	Laser group improved VHI; however, no differences in the mean improvement between control groups	⨁⨁◯◯ Low	Important
Quality of life (follow up: mean 6 months; assessed with: SF‐12/EORTC QLQ‐BR45)									
2	Randomized trials	Serious^a^	Serious^b^	Not serious	Not serious	None	Laser group was no improvement in quality of life based on Short‐Form 12 test. Laser group was improvement in QoL domains based on EORTC QLQ‐BR45 questionnaire: systemic therapy side effects (*P* = 0.034), endocrine therapy symptoms (*P* = 0.033), mucosis symptoms (*P* = 0.005), sexual enjoyment (*P* = 0.023), and endocrine sexual symptoms (*P <* 0.001). However, worsening in sexual functioning domain (*P* = 0.004) and no significant differences in the mean improvement in all QoL domains between groups	⨁⨁◯◯ Low	Important

Abbreviations: EORTC‐QLQ‐BR45, European Organization for Research and Treatment of Cancer quality of life questionnaire; NRS, numeric rating scale; SF‐12, Short‐Form 12; VAS, visual analog scale; VHI, Vaginal Health Index.

^a^ Bias deviatons from intended intervention and bias for measurement of the outcome for lack of blinded.

^b^ High heterogeneity due to different: measurement scales, physical intravaginal methods, comparator groups, analysis of different outcome measures.

^c^ The outcomes are variable.

## 4 DISCUSSION

Dyspareunia was the main outcome assessed in the three trials,^25^, ^26^, ^27^ showing a reduction in symptom intensity with physical energy treatment, which positively impacted patients' well‐being, with effects lasting up to 6 months. However, these improvements did not reach statistical significance between the intervention and control groups.

Results from the longest follow up, published within 2 years by Quick et al.,^28^ showed no statistically significant difference in VAS scores for dyspareunia with CO_2_ laser treatment. However, data from a retrospective study that included both healthy women and BCS demonstrated statistically significant improvements in dyspareunia and vaginal dryness using the same energy source.^29^

For VHI results, an improvement was observed in the laser group. However, the latter was not statistically significant among groups.^25^, ^27^ Although these differences did not reach statistical significance, they may still hold clinical relevance, as even modest symptom improvements can meaningfully enhance patients' quality of life and overall well‐being.

A retrospective analysis of BCS using CO_2_ laser showed an improvement of VHI significantly constant at every laser session after 3, 6, and 12 months.^29^ Recent data in an RCT using radiofrequency compared with vaginal estrogen, in postmenopausal women, showed a significant difference between groups, with greater VHI improvement in the radiofrequency group.^30^ Concordant results with Er:YAG laser, including postmenopausal women with a history of breast cancer, found an improvement in vaginal health, as demonstrated by an increased overall score.^29^, ^31^

Sexual function was evaluated only by Mension et al.^27^ who reported improvement in sexually active women. However, this improvement was not observed between groups. Similarly, a single‐arm study with CO_2_ laser in BCS showed an improvement in sexual function in each of the six domains of the FSFI at 12 weeks (*P <* 0.001).^32^ Another prospective study with Er:YAG laser in BCS, showed a statistically significant improvement in Short Personal Experiences Questionnaire (SPEQ) sexual function score, between pretreatment and post‐treatment (*P* = 0.04).^31^ Similarly, an overview of nine systematic reviews of postmenopausal women without a history of breast cancer demonstrated an improvement in sexual function in most studies with physical energy.^33^

All trials consistently indicated that laser treatment is safe. None of the studies reports consensus on the number of treatment sessions or recommended protocols. One of the confounding factors is the variation in protocols implemented at each energy. During data selection, we identified one study conducted by Fidecicchi et al.^34^ that involved a randomized comparison of two laser treatment protocols in BCS experiencing dyspareunia. This study showed a reduction in superficial dyspareunia in hyperstack mode compared with the standard protocol. However, because of the lack of a comparative group, it could not be included in the review.^34^

In conclusion, physical energy methods appear to be safe for the short‐term treatment of GSM in BCS. However, the limited number of blinded clinical trials makes the efficacy results uncertain. Further high‐quality RCTs with long‐term follow up are needed.

The strength of this review lies in its novelty as one of the first to focus on BCS and to include only RCTs. However, some limitations should be noted, including the small number of studies, small sample sizes, and short follow‐up periods. The methodologic and outcome heterogeneity among studies, with variations in physical energy types, protocols, control groups, assessed outcomes, and follow‐up periods, renders meta‐analyses unsuitable and complicates the interpretation of the efficacy of physical energy treatments. Numerous studies are currently underway worldwide to further confirm the effectiveness of physical energy treatments for GSM in BCS.

Intravaginal physical energy has the potential to serve as a non‐hormonal therapy option for relieving symptoms of GSM in BCS. However, there are currently no studies with long‐term follow up, homogeneous outcomes, or sham‐controlled groups, which limits the certainty of the evidence and the adoption of this intervention in clinical practice.

To date, there is a continued need for alternative treatments to address this condition. Although certain therapies may be costly, they could be considered viable options because of their ease of application. However, daily administration of hormones and lubricants often results in low adherence rates, primarily as a result of the difficulties of maintaining long‐term compliance.^35^, ^36^ This underscores the need for more accessible and sustainable therapeutic solutions that patients can easily integrate into their daily routines.

## AUTHOR CONTRIBUTIONS

NS and AKG designed the study. NS, ACAS, and ACQdA screened the abstracts for inclusion in the study. NS, NRA, JDdASC, and MLN analyzed the data. NS, ACAS, KSdM, and AKG coordinated discussions and helped interpret the data. The grade was assessed by NS, ACAS, and ACQdA. NS, ACAS, and AKG drafted the manuscript, which was then critically reviewed by all authors. All authors approved the final manuscript.

## CONFLICT OF INTEREST STATEMENT

The authors have no conflicts of interest.

## Supporting information


**Appendix S1.** Peer review of electronic search strategies.

**Table S1.** List of clinical trial records registered on ClinicalTrials.gov currently in the patient recruitment phase.

## Data Availability

Data sharing is not applicable to this article as no new data were created or analyzed in this study.

## References

[1] Davis SR , Panjari M , Robinson PJ , Fradkin P , Bell RJ . Menopausal symptoms in breast cancer survivors nearly 6 years after diagnosis. Menopause. 2014;21(10):1075‐1081. doi:10.1097/GME.0000000000000219 24618765

[2] Cook ED , Iglehart EI , Baum G , Schover LL , Newman LL . Missing documentation in breast cancer survivors: genitourinary syndrome of menopause. Menopause. 2017;24(12):1360‐1364. doi:10.1097/GME.0000000000000926 28640166 PMC5709152

[3] Meriggiola MC , Villa P , Maffei S , et al. Vulvovaginal atrophy in women with and without a history of breast cancer: baseline data from the PatiEnt satisfactiON studY (PEONY) in Italy. Maturitas. 2024;183:107950. doi:10.1016/j.maturitas.2024.107950 38462385

[4] Gupta P , Sturdee DW , Palin SL , et al. Menopausal symptoms in women treated for breast cancer: the prevalence and severity of symptoms and their perceived effects on quality of life. Climacteric. 2006;9(1):49‐58. doi:10.1080/13697130500487224 16428125

[5] Wei T , Li X , Qiang W , et al. Menopausal symptoms in breast cancer patients receiving adjuvant endocrine therapy and their relationships with health‐promoting behaviors and social support. Menopause. 2023;30(3):289‐295. doi:10.1097/GME.0000000000002130 36728825

[6] Santen RJ , Stuenkel CA , Davis SR , Pinkerton JV , Gompel A , Lumsden MA . Managing menopausal symptoms and associated clinical issues in breast cancer survivors. J Clin Endocrinol Metab. 2017;102(10):3647‐3661. doi:10.1210/jc.2017-01138 28934376

[7] The 2022 hormone therapy position statement of the North American Menopause Society advisory panel. The 2022 hormone therapy position statement of the North American Menopause Society. Menopause. 2022;29(7):767‐794. doi:10.1097/GME.0000000000002028 35797481

[8] Lubián López DM . Management of genitourinary syndrome of menopause in breast cancer survivors: an update. World J Clin Oncol. 2022;13(2):71‐100. doi:10.5306/wjco.v13.i2.71 35316932 PMC8894268

[9] Spronk I , Korevaar JC , Schellevis FG , Albreht T , Burgers JS . Evidence‐based recommendations on care for breast cancer survivors for primary care providers: a review of evidence‐based breast cancer guidelines. BMJ Open. 2017;7(12):e015118. doi:10.1136/bmjopen-2016-015118 PMC5728293 29237652

[10] Krishnamurthy J , Tandra PK . Genitourinary syndrome of menopause in breast cancer survivors: a common complication with effective treatment strategies. J Oncol Pract. 2019;15(7):373‐374. doi:10.1200/JOP.19.00297 31291560

[11] Sarmento ACA , Lírio JF , Medeiros KS , et al. Physical methods for the treatment of genitourinary syndrome of menopause: a systematic review. Int J Gynaecol Obstet. 2021;153(2):200‐219. doi:10.1002/ijgo.13561 33354773

[12] Pessoa LL , Souza AT , Sarmento AC , et al. Laser therapy for genitourinary syndrome of menopause: systematic review and meta‐analysis of randomized controlled trial. Rev Bras Ginecol Obstet. 2024;46:e‐rbgo38. doi:10.61622/rbgo/2024rbgo38 PMC11460430 39381344

[13] Filippini M , Porcari I , Ruffolo AF , et al. CO2‐laser therapy and genitourinary syndrome of menopause: a systematic review and meta‐analysis. J Sex Med. 2022;19(3):452‐470. doi:10.1016/j.jsxm.2021.12.010 35101378

[14] Jha S , Wyld L , Krishnaswamy PH . The impact of vaginal laser treatment for genitourinary syndrome of menopause in breast cancer survivors: a systematic review and meta‐analysis. Clin Breast Cancer. 2019;19(4):e556‐e562. doi:10.1016/j.clbc.2019.04.007 31227415

[15] Knight C , Logan V , Fenlon D . A systematic review of laser therapy for vulvovaginal atrophy/genitourinary syndrome of menopause in breast cancer survivors. Ecancermedicalscience. 2019;13:988. doi:10.3332/ecancer.2019.988 32010212 PMC6974376

[16] Mension E , Alonso I , Castelo‐Branco C . Genitourinary syndrome of menopause: current treatment options in breast cancer survivors—systematic review. Maturitas. 2021;143:47‐58. doi:10.1016/j.maturitas.2020.08.010 33308636

[17] Higgins JPT , Thomas J , Chandler J , et al., eds. Cochrane handbook for systematic reviews of interventions version 6.5 (updated august 2024). Cochrane; 2024. www.training.cochrane.org/handbook

[18] Page MJ , McKenzie JE , Bossuyt PM , et al. The PRISMA 2020 statement: an updated guideline for reporting systematic reviews. BMJ. 2021;372:n71. doi:10.1136/bmj.n71 33782057 PMC8005924

[19] Serquiz N , Sarmento ACA , Almeida NR , et al. Laser and radiofrequency for treating genitourinary syndrome of menopause in breast cancer survivors: a systematic review and meta‐analysis protocol. BMJ Open. 2023;13(11):e075841. doi:10.1136/bmjopen-2023-075841 PMC10649472 37949628

[20] Lukacz ES , Lawrence JM , Burchette RJ , Luber KM , Nager CW , Buckwalter JG . The use of visual analog scale in urogynecologic research: a psychometric evaluation. Am J Obstet Gynecol. 2004;191(1):165‐170. doi:10.1016/j.ajog.2004.04.047 15295359

[21] Wiegel M , Meston C , Rosen R . The female sexual function index (FSFI): cross‐validation and development of clinical cutoff scores. J Sex Marital Ther. 2005;31(1):1‐20. doi:10.1080/00926230590475206 15841702

[22] Weber MA , Limpens J , Roovers JP . Assessment of vaginal atrophy: a review. Int Urogynecol J. 2015;26(1):15‐28. doi:10.1007/s00192-014-2464-0 25047897

[23] Sterne JAC , Savović J , Page MJ , et al. RoB 2: a revised tool for assessing risk of bias in randomised trials. BMJ. 2019;366:l4898. doi:10.1136/bmj.l4898 31462531

[24] Guyatt GH , Thorlund K , Oxman AD , et al. GRADE guidelines: 13. Preparing summary of findings tables and evidence profiles‐continuous outcomes. J Clin Epidemiol. 2013;66(2):173‐183. doi:10.1016/j.jclinepi.2012.08.001 23116689

[25] Gold D , Nicolay L , Avian A , et al. Vaginal laser therapy versus hyaluronic acid suppositories for women with symptoms of urogenital atrophy after treatment for breast cancer: a randomized controlled trial. Maturitas. 2023;167:1‐7. doi:10.1016/j.maturitas.2022.08.013 36279690

[26] Fernandes MFR , Bianchi‐Ferraro AMHM , Sartori MGF , et al. CO 2 laser, radiofrequency, and promestriene in the treatment of genitourinary syndrome of menopause in breast cancer survivors: a histomorphometric evaluation of the vulvar vestibule. Menopause. 2023;30(12):1213‐1220. doi:10.1097/GME.0000000000002274 37963315

[27] Mension E , Alonso I , Anglès‐Acedo S , et al. Effect of fractional carbon dioxide vs. sham laser on sexual function in survivors of breast cancer receiving aromatase inhibitors for genitourinary syndrome of menopause: the LIGHT randomized clinical trial. JAMA Netw Open. 2023;6(2):e2255697. doi:10.1001/jamanetworkopen.2022.55697 36763359 PMC9918877

[28] Quick AM , Hundley A , Evans C , et al. Long‐Term Follow‐Up of Fractional CO2 Laser Therapy for Genitourinary Syndrome of Menopause in Breast Cancer Survivors. J Clin Med. 2022;11(3):774. doi:10.3390/jcm11030774 35160226 PMC8836519

[29] Siliquini GP , Bounous VE , Novara L , Giorgi M , Bert F , Biglia N . Fractional CO_2_ vaginal laser for the genitourinary syndrome of menopause in breast cancer survivors. Breast J. 2021;27(5):448‐455. doi:10.1111/tbj.14211 33728801

[30] Sarmento ACA , Fernandes FS , Maia RR , et al. Microablative fractional radiofrequency for sexual dysfunction and vaginal Trophism: a randomized clinical trial. Clinics (Sao Paulo). 2023;78:100293. doi:10.1016/j.clinsp.2023.100293 37839177 PMC10589764

[31] Arêas F , Valadares ALR , Conde DM , Costa‐Paiva L . The effect of vaginal erbium laser treatment on sexual function and vaginal health in women with a history of breast cancer and symptoms of the genitourinary syndrome of menopause: a prospective study. Menopause. 2019;26(9):1052‐1058. doi:10.1097/GME.0000000000001353 31453969

[32] Pearson A , Booker A , Tio M , Marx G . Vaginal CO2 laser for the treatment of vulvovaginal atrophy in women with breast cancer: LAAVA pilot study. Breast Cancer Res Treat. 2019;178(1):135‐140. doi:10.1007/s10549-019-05384-9 31377895

[33] Sarmento ACA , de Araújo Santos Camargo JD , Freitas CL , Medeiros KS , Costa APF , Gonçalves AK . Physical energies for the management of genitourinary syndrome of menopause: an overview of a systematic review and network meta‐analysis. Int J Gynaecol Obstet. 2024;166(1):163‐172. doi:10.1002/ijgo.15304 38102987

[34] Fidecicchi T , Gaspar A , Gambacciani M . Superficial dyspareunia treatment with hyperstacking of erbium:yttrium‐aluminum‐garnet SMOOTH laser: a short‐term, pilot study in breast cancer survivors. Menopause. 2023;30(2):174‐178. doi:10.1097/GME.0000000000002118 36696641

[35] Sánchez‐Borrego R , de Diego Pérez de Zabalza MV , Alfageme Gullón MJ , et al. Satisfaction and medication adherence in women with vulvovaginal atrophy: the CRETA study. Climacteric. 2023;26(5):437‐444. doi:10.1080/13697137.2023.2190508 37017707

[36] Sarmento ACA , Kamilos MF , Costa APF , Vieira‐Baptista P , Eleutério J Jr , Gonçalves AK . Use of moisturizers and lubricants for vulvovaginal atrophy. Front Reprod Health. 2021;3:781353. doi:10.3389/frph.2021.781353 36303977 PMC9580673

