# Supplementary material for: Laser and radiofrequency for treating genitourinary syndrome of menopause in breast cancer survivors: A systematic review of randomized controlled trial
Source: Int J Gynaecol Obstet. 2025 Nov 15;173(1):99–108. doi: 10.1002/ijgo.70665 (PMC12988403; doi:10.1002/ijgo.70665)
Supplement: Supplementary file 2 — Table S1. List of clinical trial records registered on ClinicalTrials.gov currently in the patient recruitment phase. [file IJGO-173-99-s002.docx]

Table S1: List of Clinical Trial records registered on ClinicalTrials.gov currently in the patient recruitment phase.

| **Study Title** | **NCT Number** | **Status** | **Conditions** | **Interventions** |
| --- | --- | --- | --- | --- |
| [LASER and Radiofrequency for Treatment of Vaginal Vulvar Atrophy (VVA) in Women Treated for Breast Cancer (EPMLARF-arm2)](https://clinicaltrials.gov/study/NCT04081805?intr=(Breast%20neoplasm%20OR%20Breast%20cancer%20OR%20female%20cancer%20OR%20cancer%20survivors%20OR%20menopause%20OR%20postmenopause)%20AND%20(Laser%20Therapy%20OR%20laser,%20Erbium%20YAG%20OR%20La-sers,%20CO2%20OR%20Laser,%20Carbon%20Dioxide%20OR%20Radiofrequency%20Therapy%20OR%20Radiofre-quency%20therapies%20OR%20Therapy,%20Radiofrequency%20OR%20Radio-frequency%20therapy)%20AND%20(genitourinary%20syndrome%20of%20menopause%20OR%20Atrophic%20Vaginitis%20OR%20Atrophy%20OR%20dyspareunia%20OR%20sexual%20health%20OR%20sexual%20dysfunction,%20physiological%20OR%20dysuria%20OR%20quality%20of%20life)&aggFilters=status:not%20rec&viewType=Table&rank=1) | **NCT04081805**  **Brazil** | Recruiting | Vulvovaginal atrophy moderate and severe BCS treated. | Device: CO2 Laser  Device: Micro Ablative Radiofrequency  Drug: Promestriene |
| [The Effect Of Fractional CO2 Laser Therapy On Cancer Survivors With Genitourinary Syndrome Of Menopause (GSM)](https://clinicaltrials.gov/study/NCT06318052?intr=(Breast%20neoplasm%20OR%20Breast%20cancer%20OR%20female%20cancer%20OR%20cancer%20survivors%20OR%20menopause%20OR%20postmenopause)%20AND%20(Laser%20Therapy%20OR%20laser,%20Erbium%20YAG%20OR%20La-sers,%20CO2%20OR%20Laser,%20Carbon%20Dioxide%20OR%20Radiofrequency%20Therapy%20OR%20Radiofre-quency%20therapies%20OR%20Therapy,%20Radiofrequency%20OR%20Radio-frequency%20therapy)%20AND%20(genitourinary%20syndrome%20of%20menopause%20OR%20Atrophic%20Vaginitis%20OR%20Atrophy%20OR%20dyspareunia%20OR%20sexual%20health%20OR%20sexual%20dysfunction,%20physiological%20OR%20dysuria%20OR%20quality%20of%20life)&aggFilters=status:not%20rec&viewType=Table&rank=3) | **NCT06318052**  **Germany** | Recruiting | Non-metastatic BCS (M0, ≤Stage IIIA). Sexually active women with severity and persist dyspareunia and/or vaginal dryness. | Device: The FemTouch delivery system  Drug: Gynomunal gel treatment |
| [Testing Laser Therapy for Treatment of Vaginal Dryness in Survivors of Breast Cancer, The Revitalize Trial](https://clinicaltrials.gov/study/NCT05379153?intr=(Breast%20neoplasm%20OR%20Breast%20cancer%20OR%20female%20cancer%20OR%20cancer%20survivors%20OR%20menopause%20OR%20postmenopause)%20AND%20(Laser%20Therapy%20OR%20laser,%20Erbium%20YAG%20OR%20La-sers,%20CO2%20OR%20Laser,%20Carbon%20Dioxide%20OR%20Radiofrequency%20Therapy%20OR%20Radiofre-quency%20therapies%20OR%20Therapy,%20Radiofrequency%20OR%20Radio-frequency%20therapy)%20AND%20(genitourinary%20syndrome%20of%20menopause%20OR%20Atrophic%20Vaginitis%20OR%20Atrophy%20OR%20dyspareunia%20OR%20sexual%20health%20OR%20sexual%20dysfunction,%20physiological%20OR%20dysuria%20OR%20quality%20of%20life)&aggFilters=status:not%20rec&viewType=Table&rank=4) | **NCT05379153 United States** | Not yet recruiting | BCS treated with curative intent; Vaginal dryness with or without dyspareunia of at least moderate severity. | Device: CO2 laser  Device: Sham Intervention |
| [Combination of the Vaginal Laser With a Moisturizing and Repairing Niosomal Gel in the Treatment of Genitourinary Syndrome in Oncology Patients](https://clinicaltrials.gov/study/NCT06508788?intr=(Breast%20neoplasm%20OR%20Breast%20cancer%20OR%20female%20cancer%20OR%20cancer%20survivors%20OR%20menopause%20OR%20postmenopause)%20AND%20(Laser%20Therapy%20OR%20laser,%20Erbium%20YAG%20OR%20La-sers,%20CO2%20OR%20Laser,%20Carbon%20Dioxide%20OR%20Radiofrequency%20Therapy%20OR%20Radiofre-quency%20therapies%20OR%20Therapy,%20Radiofrequency%20OR%20Radio-frequency%20therapy)%20AND%20(genitourinary%20syndrome%20of%20menopause%20OR%20Atrophic%20Vaginitis%20OR%20Atrophy%20OR%20dyspareunia%20OR%20sexual%20health%20OR%20sexual%20dysfunction,%20physiological%20OR%20dysuria%20OR%20quality%20of%20life)&aggFilters=status:not%20rec&viewType=Table&rank=5) | **NCT06508788**  **Spain** | Recruiting | History of neoplasia not eligible for treatment with hormonal therapy GSM. | Device: CO2 laser cohort |
| [Use of Fractional CO2 Laser Versus Erbium:YAG Laser for the Treatment of GSM in Patients Using Aromatase Inhibitors](https://clinicaltrials.gov/study/NCT05713435?intr=(Breast%20neoplasm%20OR%20Breast%20cancer%20OR%20female%20cancer%20OR%20cancer%20survivors%20OR%20menopause%20OR%20postmenopause)%20AND%20(Laser%20Therapy%20OR%20laser,%20Erbium%20YAG%20OR%20La-sers,%20CO2%20OR%20Laser,%20Carbon%20Dioxide%20OR%20Radiofrequency%20Therapy%20OR%20Radiofre-quency%20therapies%20OR%20Therapy,%20Radiofrequency%20OR%20Radio-frequency%20therapy)%20AND%20(genitourinary%20syndrome%20of%20menopause%20OR%20Atrophic%20Vaginitis%20OR%20Atrophy%20OR%20dyspareunia%20OR%20sexual%20health%20OR%20sexual%20dysfunction,%20physiological%20OR%20dysuria%20OR%20quality%20of%20life)&aggFilters=status:not%20rec&viewType=Table&rank=6) | **NCT05713435**  **Belgium** | Recruiting | BCS Moderate to Severe symptoms of GSM. | Device: CO2 laser  Device: YAG laser  Device: Sham comparator |
| [Evaluation of Innovative Therapeutic Approaches of Vaginal and Sexual Dysfunction After Breast Cancer Treatment](https://clinicaltrials.gov/study/NCT04713917?intr=(Breast%20neoplasm%20OR%20Breast%20cancer%20OR%20female%20cancer%20OR%20cancer%20survivors%20OR%20menopause%20OR%20postmenopause)%20AND%20(Laser%20Therapy%20OR%20laser,%20Erbium%20YAG%20OR%20La-sers,%20CO2%20OR%20Laser,%20Carbon%20Dioxide%20OR%20Radiofrequency%20Therapy%20OR%20Radiofre-quency%20therapies%20OR%20Therapy,%20Radiofrequency%20OR%20Radio-frequency%20therapy)%20AND%20(genitourinary%20syndrome%20of%20menopause%20OR%20Atrophic%20Vaginitis%20OR%20Atrophy%20OR%20dyspareunia%20OR%20sexual%20health%20OR%20sexual%20dysfunction,%20physiological%20OR%20dysuria%20OR%20quality%20of%20life)&aggFilters=status:not%20rec&viewType=Table&rank=8) | **NCT04713917**  **France** | Recruiting | Non-metastatic breast cancer VVA. | Device: Hyaluronique Acid Gel  Device: Laser CO2  Device: Hyaluronique Acid Injection |
| [A Laser and Topical Treatment Combination in the Vulvo-vaginal Atrophy Management in Breast Cancer Patients.](https://clinicaltrials.gov/study/NCT05585476?intr=(Breast%20neoplasm%20OR%20Breast%20cancer%20OR%20female%20cancer%20OR%20cancer%20survivors%20OR%20menopause%20OR%20postmenopause)%20AND%20(Laser%20Therapy%20OR%20laser,%20Erbium%20YAG%20OR%20La-sers,%20CO2%20OR%20Laser,%20Carbon%20Dioxide%20OR%20Radiofrequency%20Therapy%20OR%20Radiofre-quency%20therapies%20OR%20Therapy,%20Radiofrequency%20OR%20Radio-frequency%20therapy)%20AND%20(genitourinary%20syndrome%20of%20menopause%20OR%20Atrophic%20Vaginitis%20OR%20Atrophy%20OR%20dyspareunia%20OR%20sexual%20health%20OR%20sexual%20dysfunction,%20physiological%20OR%20dysuria%20OR%20quality%20of%20life)&aggFilters=status:not%20rec&viewType=Table&rank=12) | **NCT05585476**  **Spain** | Not yet recruiting | BCS Female VVA. | Device: C02 microablative laser  Combination Product: Regenerative topical treatment |

Legend: VVA: Vaginal Vulvar Atrophy; GSM: Genitourinary Syndrome of Menopause; BCS: Breast cancer survivors; CO2 Laser, thermoablative fractional CO2 laser; YAG Laser, YAG, Yttrium- aluminum- garnet
