# Supplementary material for: Laser and radiofrequency for treating genitourinary syndrome of menopause in breast cancer survivors: A systematic review of randomized controlled trial
Source: Int J Gynaecol Obstet. 2025 Nov 15;173(1):99–108. doi: 10.1002/ijgo.70665 (PMC12988403; doi:10.1002/ijgo.70665)
Supplement: Supplementary file 1 — Appendix S1. Peer review of electronic search strategies. [file IJGO-173-99-s001.docx]

# **Peer Review of Electronic Search Strategies**

##### *PRESS Guideline*—Search Submission & Peer Review Assessment

##### SEARCH SUBMISSION: THIS SECTION TO BE FILLED IN BY THE SEARCHER

| Searcher: Nicoli Serquiz de Azevedo | Email: biblioteca@ccs.ufrn.br  Librarians: Ana Cristina |
| --- | --- |
| Date submitted: 20/06/2024 | Date requested by: 01/07/2024 |

**Systematic Review Title:**

Laser and radiofrequency for treating genitourinary syndrome of menopause in breast cancer survivors: a systematic review and meta-analysis

##### This search strategy

| x | My PRIMARY (core) database strategy — First time submitting a strategy for search questioned database |
| --- | --- |
|  | My PRIMARY (core) strategy — Follow-up review NOT the first time submitting a strategy for search question and database. If this is a response to peer review, itemize the changes made to there view suggestions |
|  | SECONDARY search strategy— First time submitting a strategy for search question and database |
|  | SECONDARY search strategy — NOT the first time submitting a strategy for search question and database. If this is a response to peer review, itemize the changes made to the review suggestions |

**Database**

(i.e.,MEDLINE,CINAHL…):*[mandatory]*

PubMed, Scopus, Web of Science, Cochrane Central Register of Controlled Trials, clinical trials databases; SciELO; LILACS

##### Interface

(i.e.,Ovid,EBSCO…):*[mandatory]*

EMBASE

##### Research Question

(Describe the purpose of the search)*[mandatory]*

Are intravaginal physical energies effective and safe for the management of genitourinary syndrome of menopause (GSM) among breast cancer survivors?

##### PICO Format

(Outline the PICOs for your question — i.e., Patient, Intervention, Comparison,Outcome,and Study Design—as applicable)

| **P** | Breast cancer survivors |
| --- | --- |
| **I** | Intravaginal physical energy (laser or radiofrequency) |
| **C** | No treatment, placebo, sham, and/or other treatment |
| **O** | Improvement of vaginal health, vaginal atrophy, dryness, dyspareunia, itching, burning, dysuria, incontinence, sexual dysfunction, quality of life, and side effects |
| **S** | Randomized or quasi-randomized clinical trial |

##### Inclusion Criteria

(List criteria such as age groups, study designs, etc., to be included)*[optional]*

Randomised and quasi-randomised clinical trials that investigate both the efficacy and safety of any intravaginal physical energy (laser and radiofrequency) in non-metastatic BCSs, irrespective of age, who are still sexually active or willing to resume sexual activity with one or more GSM symptoms, will be included.

##### Exclusion Criteria

(List criteria such as study designs,date limits,etc.,to be excluded)*[optional]*

Studies involving women with recurrent or metastatic cancer, prior reconstructive pelvic surgery involving a mesh for prolapse and active genital infections (diagnosed by Gram staining and multiplex PCR) and studies that do not assess the outcomes will be excluded. Observational studies, case reports, review articles, reports and case series will also be excluded.

##### Was a search filter applied?

Yes □	Nox

**If YES, which one(s) (e.g., Cochrane RCT filter, PubMed Clinical Queries filter)?Provide the source if this is a published filter.***[mandatory If YES to previous question*—*text box]*

Other notes or comments you feel would be useful for the peer reviewer?***[optional]***

Safe and effective non-hormonal treatments are needed to improve the long-term health of women with breast cancer. Intravaginal energy-based methods (laser and radiofrequency) have recently been proposed as non-pharmacological therapeutical options for managing GSM.

Please copy and paste your search strategy here, exactly as run, including the number of hits per line. ***[mandatory]***

##### (Add more space, as necessary.)

|  | **DECS OR MESH and Synonyms** | **STRATEGY LINES** | **NUMBER OF STUDIES FOUND** |
| --- | --- | --- | --- |
| **P** | Breast Neoplasms | (“Breast Neoplasms” OR “Breast Cancer” OR “Female cancer” OR “Cancer Survivors” OR Menopause OR Postmenopause) | 592,775 |
|  | **AND** |  |  |
| **I** | Laser Therapy | (“Laser Therapy” OR Lasers OR “Erbium YAG Laser” OR “Lasers, CO2” OR “Laser, Carbon Dioxide” OR “Radiofrequency Therapy” OR “Radiofrequency therapies” OR “Therapy, Radiofrequency” OR “Radio-Frequency therapy” OR “Energy based devices”) | 403,648 |
| **C** | Therapeutics | (Therapeutics OR Therapeutic OR Therapy OR Therapies OR Treatment OR Treatments) | 14,308,522 |
|  | **AND** |  |  |
| **O** | Sexual Dysfunctions, Physiologica | (“Genitourinary syndrome of menopause” OR “Atrophic vaginitis” OR Atrophy OR dyspareunia OR “Sexual health” OR “Sexual Dysfunctions, Physiological” OR Dysuria OR “Quality of Life”) | 658,150 |

* Use the acronym adaptations as necessary.

| **DATABASE** | **STRATEGY** | **NUMBER OF STUDIES FOUND** |
| --- | --- | --- |
| PUBMED | (“Breast Neoplasms” OR “Breast Cancer” OR “Female cancer” OR “Cancer Survivors” OR Menopause OR Postmenopause) AND (“Laser Therapy” OR Lasers OR “Erbium YAG Laser” OR “Lasers, CO2” OR “Laser, Carbon Dioxide” OR “Radiofrequency Therapy” OR “Radiofrequency therapies” OR “Therapy, Radiofrequency” OR “Radio-Frequency therapy” OR “Energy based devices”) AND (Therapeutics OR Therapeutic OR Therapy OR Therapies OR Treatment OR Treatments) AND (“Genitourinary syndrome of menopause” OR “Atrophic vaginitis” OR Atrophy OR dyspareunia OR “Sexual health” OR “Sexual Dysfunctions, Physiological” OR Dysuria OR “Quality of Life”) | 392 |

| **DATABASE** | **STRATEGY** | **NUMBER OF STUDIES FOUND** |
| --- | --- | --- |
| EMBASE | (“Breast Neoplasm” OR “Breast Cancer” OR “Female cancer” OR “Cancer Survivors” OR Menopause OR Postmenopause) AND (“Laser Therapy” OR Lasers OR “Erbium YAG Laser” OR “Lasers, CO2” OR “Laser, Carbon Dioxide” OR “Radiofrequency Therapy” OR “Radiofrequency therapies” OR “Therapy, Radiofrequency” OR “Radio-Frequency therapy” OR “Energy based devices”) AND (Therapeutics OR Therapeutic OR Therapy OR Therapies OR Treatment OR Treatments) AND (“Genitourinary syndrome of menopause” OR “Atrophic vaginitis” OR Atrophy OR dyspareunia OR “Sexual health” OR “Sexual Dysfunctions, Physiological” OR Dysuria OR “Quality of Life”) | 953 |

| **DATABASE** | **STRATEGY** | **NUMBER OF STUDIES FOUND** |
| --- | --- | --- |
| Web of Science | (“Breast Neoplasm” OR “Breast Cancer” OR “Female cancer” OR “Cancer Survivors” OR Menopause OR Postmenopause) AND (“Laser Therapy” OR Lasers OR “Erbium YAG Laser” OR “Lasers, CO2” OR “Laser, Carbon Dioxide” OR “Radiofrequency Therapy” OR “Radiofrequency therapies” OR “Therapy, Radiofrequency” OR “Radio-Frequency therapy” OR “Energy based devices”) AND (Therapeutics OR Therapeutic OR Therapy OR Therapies OR Treatment OR Treatments) AND (“Genitourinary syndrome of menopause” OR “Atrophic vaginitis” OR Atrophy OR dyspareunia OR “Sexual health” OR “Sexual Dysfunctions, Physiological” OR Dysuria OR “Quality of Life”) | 469 |

| **DATABASE** | **STRATEGY** | **NUMBER OF STUDIES FOUND** |
| --- | --- | --- |
| Cochrane | (“Breast Neoplasm” OR “Breast Cancer” OR “Female cancer” OR “Cancer Survivors” OR Menopause OR Postmenopause) AND (“Laser Therapy” OR Lasers OR “Erbium YAG Laser” OR “Lasers, CO2” OR “Laser, Carbon Dioxide” OR “Radiofrequency Therapy” OR “Radiofrequency therapies” OR “Therapy, Radiofrequency” OR “Radio-Frequency therapy” OR “Energy based devices”) AND (Therapeutics OR Therapeutic OR Therapy OR Therapies OR Treatment OR Treatments) AND (“Genitourinary syndrome of menopause” OR “Atrophic vaginitis” OR Atrophy OR dyspareunia OR “Sexual health” OR “Sexual Dysfunctions, Physiological” OR Dysuria OR “Quality of Life”) | 213 |

| **DATABASE** | **STRATEGY** | **NUMBER OF STUDIES FOUND** |
| --- | --- | --- |
| LILACS | (“Breast Neoplasm” OR “Breast Cancer” OR “Female cancer” OR “Cancer Survivors” OR Menopause OR Postmenopause) AND (“Laser Therapy” OR Lasers OR “Erbium YAG Laser” OR “Lasers, CO2” OR “Laser, Carbon Dioxide” OR “Radiofrequency Therapy” OR “Radiofrequency therapies” OR “Therapy, Radiofrequency” OR “Radio-Frequency therapy” OR “Energy based devices”) AND (Therapeutics OR Therapeutic OR Therapy OR Therapies OR Treatment OR Treatments) AND (“Genitourinary syndrome of menopause” OR “Atrophic vaginitis” OR Atrophy OR dyspareunia OR “Sexual health” OR “Sexual Dysfunctions, Physiological” OR Dysuria OR “Quality of Life”) | 0 |
| **DATABASE** | **STRATEGY** | **NUMBER OF STUDIES FOUND** |
| SciELO | (“Breast Neoplasm” OR “Breast Cancer” OR “Female cancer” OR “Cancer Survivors” OR Menopause OR Postmenopause) AND (“Laser Therapy” OR Lasers OR “Erbium YAG Laser” OR “Lasers, CO2” OR “Laser, Carbon Dioxide” OR “Radiofrequency Therapy” OR “Radiofrequency therapies” OR “Therapy, Radiofrequency” OR “Radio-Frequency therapy” OR “Energy based devices”) AND (Therapeutics OR Therapeutic OR Therapy OR Therapies OR Treatment OR Treatments) AND (“Genitourinary syndrome of menopause” OR “Atrophic vaginitis” OR Atrophy OR dyspareunia OR “Sexual health” OR “Sexual Dysfunctions, Physiological” OR Dysuria OR “Quality of Life”) | 0 |

| **DATABASE** | **STRATEGY** | **NUMBER OF STUDIES FOUND** |
| --- | --- | --- |
| **Scopus** | "Breast Neoplasm" OR "Breast Cancer" OR "Female cancer" OR "Cancer Survivors" OR Menopause OR Postmenopause AND "Laser Therapy" OR "Lasers, Erbium YAG Laser" OR “Lasers, CO2" OR "Laser, Carbon Dioxide" OR "Radiofrequency Therapy" OR "Radiofrequency therapies" OR "Therapy, Radiofrequency" OR "Radiofrequency therapy” OR "Energy based devices" AND "Genitourinary syndrome of menopause" OR "Atrophic vaginitis" OR "Atrophy"OR "Dyspareunia" OR "Sexual health" OR "Sexual dysfunction, Physiological" OR "Dysuria" OR "Quality of Life" | 459 |

| **DATABASE** | **STRATEGY** | **NUMBER OF STUDIES FOUND** |
| --- | --- | --- |
| [**ClinicalTrials.gov**](http://ClinicalTrials.gov) | **Condiction/Disease:**  (“Breast Neoplasm” OR “Breast Cancer” OR “Female cancer” OR “Cancer Survivors” OR Menopause OR Postmenopause)  **Other terms:**(“Genitourinary syndrome of menopause” OR “Atrophic vaginitis” OR Atrophy OR dyspareunia OR “Sexual health” OR “Sexual Dysfunctions, Physiological” OR Dysuria OR “Quality of Life”)  **Intrvention/Treatment:** (“Laser Therapy” OR Lasers OR “Erbium YAG Laser” OR “Lasers, CO2” OR “Laser, Carbon Dioxide” OR “Radiofrequency Therapy” OR “Radiofrequency therapies” OR “Therapy, Radiofrequency” OR “Radio-Frequency therapy” OR “Energy based devices”) | 103 |

**PEER REVIEW ASSESSMENT:THIS SECTION TO BE FILLED IN BY THE REVIEWER**

| **Reviewer:** | **Email:biblioteca@ccs.ufrn.br** | | **Date completed:** |
| --- | --- | --- | --- |
| **1.TRANSLATION** | | | |
|  | A.Norevisions | **□** |  |
|  | B.Revision(s)suggested | **□** |  |
|  | C.Revision(s)required | **□** |  |

If“B”or“C,”please provide an explanation or example:

| **2.BOOLEAN AND PROXIMITY OPERATORS** | | | |
| --- | --- | --- | --- |
|  | A.Norevisions | **□** |  |
|  | B.Revision(s)suggested | **□** |  |
|  | C.Revision(s)required | **□** |  |

If“B”or“C,”please provide an explanation or example:

| **3.SUBJECT HEADINGS** | | | |
| --- | --- | --- | --- |
|  | A.Norevisions | **□** |  |
|  | B.Revision(s)suggested | **□** |  |
|  | C.Revision(s)required | **□** |  |

If“B”or“C,”please provide an explanation or example:

| **4.TEXT WORD SEARCHING** | | | |
| --- | --- | --- | --- |
|  | A.Norevisions | **□** |  |
|  | B.Revision(s)suggested | **□** |  |
|  | C.Revision(s)required | **□** |  |

If“B”or“C”,please provide an explanation or example:

| **5.SPELLING,SYNTAX,ANDLINENUMBERS** | | | |
| --- | --- | --- | --- |
|  | A.Norevisions | **□** |  |
|  | B.Revision(s)suggested | **□** |  |
|  | C.Revision(s)required | **□** |  |

If“B”or“C,”please provide an explanation or example:

| **6.LIMITSANDFILTERS** | | | |
| --- | --- | --- | --- |
|  | A.Norevisions | **□** |  |
|  | B.Revision(s)suggested | **□** |  |
|  | C.Revision(s)required | **□** |  |

If“B”or“C,”please provide an explanation or example:

| **7.OVERALL EVALUATION(Note:Ifoneormore“revisionrequired”isnotedabove,the**  **responsebelowmustbe“revisionsrequired”.)** | | | |
| --- | --- | --- | --- |
|  | A.Norevisions | **□** |  |
|  | B.Revision(s)suggested | **□** |  |
|  | C.Revision(s)required | **□** |  |

Additional comments:
